# Effects of cytotoxin-associated gene A (CagA) positive Helicobacter pylori infection on anti-platelet glycoprotein antibody producing B cells in patients with primary idiopathic thrombocytopenic purpura (ITP)

**DOI:** 10.12669/pjms.311.6409

**Published:** 2015

**Authors:** Yuan-Shan Cheng, Li-Ping Kuang, Chun-Lan Zhuang, Jia-Dian Jiang, Man Shi

**Affiliations:** 1 Yuan-Shan Cheng, MD, Department of Hematology, The First Affiliated Hospital of Shantou University Medical College, Guangdong, China, 515000.; 2 Li-Ping Kuang, MD, Department of Pathology, Shantou Central Hospital, Guangdong, China, 515000.; 3 Chun-Lan Zhuang, MD, Department of Hematology, The First Affiliated Hospital of Shantou University Medical College, Guangdong, China, 515000.; 4 Jia-Dian Jiang, MD, Department of Hematology, The First Affiliated Hospital of Shantou University Medical College, Guangdong, China, 515000.; 5 Man Shi, MD, Department of Hematology, The First Affiliated Hospital of Shantou University Medical College, Guangdong, China, 515000.

**Keywords:** Idiopathic thrombocytopenic purpura, Helicobacter pylori, Cytotoxin-associated gene A, Anti-glycoprotein antibody

## Abstract

**Objective:**

To explore the effects of cytotoxin-associated gene A (CagA) positive Helicobacter pylori (H. pylori or HP) infection on circulating B cells producing specific platelet glycoprotein antibodies and the association between therapeutic outcomes in primary idiopathic thrombocytopenic purpura (ITP) patients.

**Methods:**

A total of 76 newly diagnosed primary ITP patients were included in the study which was conducted at the first affiliated hospital of Shantou University Medical college, in Shantou city China, between January 2013 and January 2014. These patients were tested for H. pylori infection by ^13^C urea breath test and for anti-CagA antibody in H. pylori positive cases by enzyme-linked immunosorbent assay (ELISA) method. Anti-GPIb and anti-GPIIb/IIIa antibody-producing B cells were measured using an enzyme-linked immunospot (ELISPOT) assay in all ITP patients and 30 controls. Anti-nuclear antibody (ANA) was also detected in ITP patients.

**Results:**

The numbers of anti-GPIIb/IIIa antibody-producing B cells in HP+CagA+ patients were higher than in HP+CagA- or HP- patients. However, anti-GPIb antibody-producing B cells were found higher in HP- patients. Analysis of treatment outcomes showed that a therapeutic response was more likely in patients presenting anti-GPIIb/IIIa B cells, but the poor response was found to be associated with anti-GPIb B cells and ANA presences.

**Conclusion:**

CagA antigen of H. *pylori* may induce anti-GPIIb/IIIa antibodies production by a molecular mimicry mechanism. Anti-GPIIb/IIIa and anti-GPIb antibody producing B Cells detection is useful for predicting treatment effects of primary ITP.

## INTRODUCTION

 Idiopathic thrombocytopenic purpura (ITP) is an autoimmune disease characterized by increased platelet destruction and impaired platelet production, both of which are mediated by anti-platelet autoantibodies. However, its pathogenesis is still unclear. The association between this disorder with hepatitis C virus (HCV), human immunodeficiency virus (HIV), and sepsis was confirmed which indicates that this disease may be caused by infection agents.^1^ In 1998, Gasbarrini, for the first time, reported that increased platelet counts were obtained after Helicobacter pylori (H. pylori) eradication in ITP patients.^2^ Later, several studies confirmed that H. pylori eradication could improve platelet counts in infected ITP patients. H. pylori eradication therapy is now recommended for H. pylori associated ITP.^3^

 The cytotoxin-associated gene A (CagA) gene codes for a 120–145kDa protein. CagA antigen is one of the major H. pylori virulence proteins. H. pylori strains can be divided into CagA positive or negative strains. In recent years, H. pylori strains that possess CagA have been proposed to have a role in the pathogenesis of ITP. One hypothesis was advanced that cross molecular mimicry between the H. pylori CagA protein and various platelets glycoprotein antigens could induce anti-glycoprotein autoantibody production cross reactive with host platelets.^4^ However there were still little data regarding the effects of CagA on specific anti-glycoprotein autoantibodies production. Therefore, we herein aimed to measure the anti-GPIIb/IIIa and anti-GPIb antibody producing B cells in primary ITP patients and correlated the results with infection H.pylori CagA genotypes and therapeutic outcomes of the patients. 

## METHODS

***Subjects: ***This study was conducted on 76 newly diagnosed primary ITP subjects (25 males and 51 females) with an average age of 35.67 years (range, 15 to 66 years) who were referred to the out-patient and in-patient clinic of hematology in the first affiliated hospital of Shantou University Medical college, in Shantou city (Guangdong province, China), between January 2013 and January 2014. The platelet counts were less than 50×10^9^/L for 2 weeks prior to study entry and patients did not display severe hemorrhagic manifestations. The diagnosis of primary ITP was confirmed by Dr. CL Zhuang according to the standard criteria proposed by the American Society of Hematology guidelines.^3^ Thirty healthy controls (average age 37.47 years, range, 17 to 65 years) matched in respect to age and sex were also included in this study. The study was approved by the ethical committee of Shantou University Medical College. All of the subjects signed the informed consent before the experiment.

***Administration and Criteria for Response: ***Patients diagnosed with primary ITP received the usual prednisone (or equivalent doses of methylprednisolone) therapy, 1-2 mg/kg/day, for 3 weeks, or combination with intravenous immunoglobulin (IVIG) 0.2-0.4 g/kg/day, for 1-5 days. Response to treatment was classified to response (platelet count ≥50,000) and no response (no rising in platelet or platelet count <50,000 or symptomatic bleeding).

***Helicobacter pylori and CagA detection: ***Thirteen carbon urea breath test (^13^C-UBT) was used to assess H. pylori infection according to a standard protocol included in the diagnosis kit (Helicobacter Test INFAI). Those found to have H. pylori infection were further tested for CagA antibody with an enzyme-linked immunosorbent assay (ELISA) kit (Human HP-Ap ELISA Kit). A result of at least 10 units per ml in the blood was considered to indicate the presence of antibody against CagA.

***Detection of B cells producing IgG anti-GPIIb/IIIa and anti-GPIb antibodies: ***Freshly peripheral blood mononuclear cells (PBMCs) isolated from heparinized peripheral blood were assayed for B cells producing IgG anti-GPIIb/IIIa and anti-GPIb antibodies, using an enzyme-linked immunospot (ELISPOT) assay. The ELISPOT assay was performed as described.^5^ Briefly, 96-well ELISPOT plates (Millipore) with polyvinylidene membranes were activated by incubation with 99.5% ethanol. After washing the plates three times with sterile PBS, the plates were coated with 30μg/ml affinity-purified human GPIIb/IIIa (Enzyme Research Laboratories) for 2 hours at RT. The plates were washed and then blocked with 1% fetal bovine serum. PBMCs suspended in RPMI with 10% fetal bovine serum were added to wells (10^5^ cells/well) in triplicate for each patient and control, and cultured at 37^0^C with 5% CO_2_ for 4 hours. The plates were washed with sterile PBS-Tween three times. The plates were then incubated for 2 hours at RT with 50μL/well (1:1000) of alkaline phosphatase-conjugated goat anti-human IgG (Sigma-Aldrich) and washed repeatedly in PBS-Tween and sterile PBS. Finally, Spots were visualized using an alkaline phosphatase conjugate substrate kit (Bio-Rad) as per manufacturer’s instruction. Recombinant human GPIbα (R&D) was used as the antigen in assay detecting B cells producing IgG anti-GPIb antibodies. Results were the mean numbers of the triplicate wells and are expressed as per 10^5^ PBMCs. The cut-off values for anti-GPIIb/IIIa and anti-GPIb antibody-producing cells were defined as the mean values plus 5 standard deviations (mean+5SD) from healthy controls.

***ANA test: ***Blood samples were collected from the patients. After blood clotted, serum was collected from the clotted samples centrifuged and kept in 2-4^0^C. ANAs were detected by indirect immunofluorescence technique on HEp-2 cells ANA kit (Euroimmune AG Company) within seven days. ANA positivity was defined as a titre ≥1:100.

***Statistical Analysis: ***Continuous variables are expressed as mean ± standard deviation (SD). Differences between two groups were checked by Student’s t test or by Mann-Whitney test (non-parametric factors). Differences in frequency between two groups were compared by the Chi-square test or Fisher’s exact test, all statistical tests were two-sided and *P* values <0.05 or 0.01 were considered statistically significant. All data were analyzed with SPSS 19.0 (SPSS Inc., Chicago, IL, USA).

## RESULTS

***Patient characteristics: ***In this study, 76 primary ITP patients and 30 health controls were enrolled overall. There was no difference in age and sex between primary ITP patients and controls.

 H. pylori infection was detected in 46 primary ITP patients by ^13^C-UBT. Twenty five out of 46 patients were CagA antibody positive. According to the results of H. pylori and CagA antibody tests, the 76 primary ITP patients were then divided into three groups: group A, H. pylori negative (HP-, 30 patients); group B, H. pylori positive but CagA negative (HP+CagA-, 21 patients) and group C, H. pylori positive CagA positive (HP+CagA+, 25 patients). There was no significant difference in age, sex, mean baseline platelet count and ANA positive rate among HP-, HP+CagA-, and HP+CagA+ groups. Patients’ characteristics in different groups were shown in Table-I.

***Quantification of anti-GPIIb/IIIa and anti-GPIb antibody B cells: ***B cells producing anti-GPIIb/IIIa and anti-GPIb antibody were detected and quantified in patients and healthy controls by ELISPOT assay. The number and positive frequencies of anti-GPIIb/IIIa producing B cells per 10^5^ PBMCs in primary ITP patients were significantly higher than in controls (5.38±3.18 VS 0.44±0.31 p<0.01; 82.89% VS 0% p<0.01). Results of primary ITP patients were further analyzed by groups as HP-, HP+CagA- and HP+CagA+. There were significantly more circulating anti-GPIIb/IIIa antibody producing B cells in HP+CagA+ group than in HP- group and HP+CagA- group (7.40±3.39 VS 4.31±2.65 p<0.01; 7.40±3.39 VS 4.49±2.61 p<0.01). The positive frequency of anti-GPIb antibody producing B cells in HP+CagA+ group were significantly higher than in HP- group (92.31%VS73.33%,p<0.05). There was no difference in anti-GPIIb/IIIa antibody-producing B cells between HP- group and HP+CagA- group (p>0.05).

**Fig.1:**
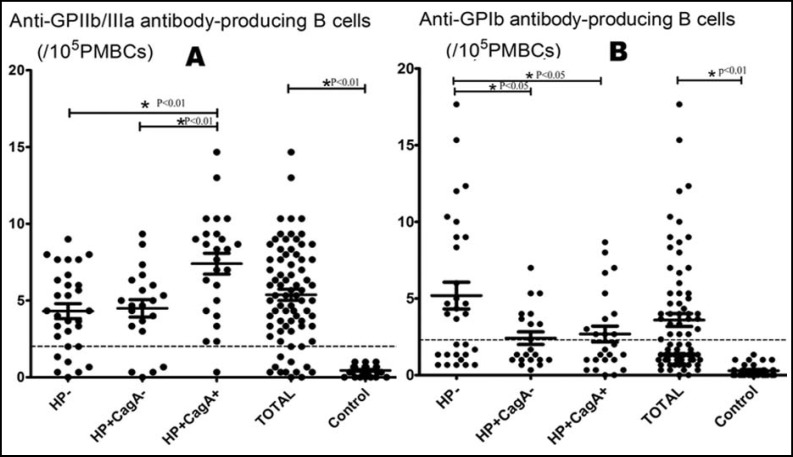
Anti-GPIIb/IIIa and anti-GPIb antibody-producing B cells in ITP patients and healthy controls

 In anti-GPIb antibody producing B cells ELISPOT assay, similarly, the number and positive frequencies were also significantly higher in primary ITP patients as compared with controls (3.60±3.66 VS 0.29±0.40 p<0.01; 48.68% VS 0% p<0.01). In contrast, H. pylori–negative patients had more antibody producing B cells response to GPIb compared with H. pylori positive patients (5.19±4.78 VS 2.41±1.88, p<0.05; 5.19±4.78 VS 2.68±2.53, p<0.05). Anti-GPIb antibody producing B cells between Hp+CagA+ and Hp+CagA- group were no statistical difference (p>0.05). Also, no statistically significant differences were found in positive frequencies of anti-GPIb antibody producing B cells among the three groups (p>0.05). Results were shown in Table-II and Fig.1.

***Therapeutic responses related to patient characteristics:*** Table-III summarizes the therapeutic responses relating to H. pylori infection status, anti-glycoprotein antibody-producing B cells, ANA results and some clinical characteristics. The results showed that a therapeutic response was more likely in patients presenting anti-GPIIb/IIIa B cells, but the poor response was found to be associated with anti-GPIb B cells and ANA presences.

## DISCUSSION

 In primary ITP, platelets are destroyed by platelet autoantibodies which bind to multiple targets on the surface of platelets. Glycoproteins IIb/IIIa and Ib in the membrane of platelets are the two most common targets.^6^ Monoclonal antibody-specific immobilization of platelet antigen (MAIPA) assay has been developed by Kiefel and colleagues for the identification of platelet alloantibodies and for platelet-specific autoantibodies detection^7^. Warner et al. reported that measurement a combination of anti-GPIIb/IIIa antibodies and anti-GPIb antibodies by MAIPA assay had a sensitivity of 66% for the diagnosis of primary ITP.^8^ Kuwana et al. detected circulating B cells producing platelet-specific autoantibodies by ELISPOT assay. They found that the ELISPOT assay was useful in the diagnosis of ITP and was more sensitive than the MAIPA assay (90% versus 66%).^9^^,^^10^ So in our study, we chose to detect anti-GPIIb/IIIa and anti-GPIb antibody producing B cells by the ELISPOT assay for the common existence of the antibodies in patients with primary ITP and the high sensitivity of the assay. In the current investigation, our results of ELISPOT were concordant with the literature data^10^, anti-GPIIb/IIIa antibody producing B cells and anti-GPIb antibody producing B cells were presented in 82.89% and 48.68% of the patients.

 Several studies have focused on the correlation between H. pylori virulence genotypes and the pathogenesis of ITP. H. pylori strains containing CagA gene were found more frequent in H. pylori associated ITP.^11^ However, there is little information available related to the autoimmunity of the patients in different H. pylori genotypes. In the present study, we have demonstrated that the anti-GPIIb/IIIa antibody producing B cells were elevated in primary ITP patients with H. pylori CagA positive infection. Our results supported the molecular mimicry hypothesis and might conclude that CagA antigen shared similar antigenic epitopes with GPIIb/IIIa instead of GPIb. Though in our results, anti-GPIb antibody producing B cells were more likely to increase in H. pylori negative primary ITP patients which showed higher ANA positivity. It is possible that ITP without H. pylori infection is caused by different mechanisms such as a systemic autoimmune condition.

 Eradication of H. pylori was reported to achieve a platelet response in approximately half of the adult ITP patients (50.3%).^12^ However, great variabilities in the efficacy of H. pylori eradication therapy from different countries were also reported.^13^^,^^14^ The efficacy of H. pylori eradication might depend on either innate immunity of the host, geography factors, or H. pylori strains.^15^^,^^16^ In the present study, we found that cases with elevated anti-GPIIb/IIIa antibody-producing B cells were more likely to respond to treatments. The presences of anti-GPIb antibody-producing B cells and positive ANA result were related to a poor therapeutic response. Our results are consistent with previous reports that the presence of platelet-associated anti-GPIb antibodies was associated with inadequate responses to corticosteroids and IVIG in ITP patients.^17^^,^^18^ Nieswandt and colleagues demonstrated that anti-GPIba monoclonal antibodies induced profound thrombocytopenia by Fc-independent mechanisms.^19^ Platelet activation and apoptosis induced by anti-GPIba antibodies are critical in the Fc-independent platelets clearance while anti-GPIIb/IIIa antibodies induced platelets clearance mainly by Fc-dependent phagocytosis. The different mechanism of platelets clearance may be at least one explanation for why anti-GPIb antibodies related to poor outcomes in some patients with ITP. Moreover, ANA presentation associates ITP with an underlying systemic autoimmune disorder, which contributes to the poor efficacy. Recently, Rocha et al. reported that platelet response of ITP after eradication of H. pylori was associated with the imbalance correction of T-helper (Th) and T regulatory (Treg) cytokines.^20^ This result indicates that the immune responses to H. pylori infected patients with ITP are different, which may be due to the difference between H. pylori strains, e.g. CagA+ and CagA- strains. The relation between the T cell cytokines and the H. pylori strains needs to be further investigated.

**Table-I:** Patients’ clinical characteristic

**Clinical characteristics**	**Primary ITP**				**Controls**
	**HP-**	**HP+CagA-**	**HP+CagA+**	**Total**	
Number	30	21	25	76	30
Sex (% female)	60%	71.43%	72%	67.10%	66.67%
Age (mean ± SD years)	35.03±10.79	36.05±13.34	36.12±9.82	35.67±9.27	37.47±14.59
Mean baseline platelet count,×10^9^/L	18.77±9.81	20.62±11.79	19.52±9.22	19.39±10.24	215.60±79.10*
ANA (% positive)	36.67%	19.05%	20.00%	26.32%	

* : Controls vs total, p<0.01

**Table-II:** Positive frequencies of anti-GPIIb/IIIa and anti-GPIb antibody-producing B cells

	**Primary ITP** **(n=76)**	**HP-** **(n=30)**	**HP+CagA-** **(n=21)**	**HP+CagA+** **(n=25)**	**Healthy** **controls** **(n=32)**
Anti-GPIIb/IIIa antibody-producing B cells alone	82.89%	73.33%	77.27%	92.31%*	0%▲
Anti-GPIb antibody-producing B cells alone	48.68%	56.67%	40.91%	42.31%	0%▲
Anti-GPIIb/IIIa antibody-producing B cells AND Anti-GPIb antibody-producing B cells	40.79%	46.67%	27.27%	42.31%	0%▲
Anti-GPIIb/IIIa antibody-producing B cells OR anti-GPIb antibody-producing B cells	90.79%	83.33%	90.91%	80.77%	0%▲

* : HP+CagA+ vs HP-, P<0.05;

▲ : Healthy controls VS primary ITP, p<0.01

**Table-III:** Therapeutic response relating to some factors

	**No response** **(NR) n=16**	**Response (R)** **n=60**	**P**
HP-	56.25%	35%	0.12
HP+CagA-	25.00%	28.00%	1.00
HP+CagA+	18.75%	36.67%	0.24
Anti-GPIIb/IIIa antibody-producing B cells present	62.50%	88.33%	0.02
Anti-GPIb antibody-producing B cells present	81.25%	40.00%	0.00
Sex (Female %)	81.25%	63.33%	0.24
Age at diagnosis (years)	32.63±7.24	36.48±12.13	0.28
Platelet count (x 109/L)	18.75±10.08	19.73±10.44	0.91
ANA	56.25%	18.33%	0.04

 Two limitations of this study should be noted. Firstly, we merely detected the anti-platelet glycoprotein antibody producing B cells in the peripheral circulation which were mostly memory B cells. We were unable to detect the glycoprotein-reactive B cells in the spleen for the reason of specimen limited. But there were studies which have demonstrated that frequencies of glycoprotein-reactive B cells were not different between the peripheral blood and spleen,^5^ so this limitation has little effect on the results that are presented in this report. Secondly, the number of patients in the study was relatively small, which might influence the accuracy of statistics. Further studies with a larger number of patients are necessary to confirm these results.

 In summary, our results have demonstrated that CagA antigen of H. pylori may induce anti-GPIIb/IIIa antibodies production by a molecular mimicry mechanism. More efforts should be put towards understanding the functional modulation of glycoprotein-reactive B cells that may be helpful to develop new strategies for the treatment of ITP.
